# Diamond FinFET without Hydrogen Termination

**DOI:** 10.1038/s41598-018-20803-5

**Published:** 2018-02-15

**Authors:** Biqin Huang, Xiwei Bai, Stephen K. Lam, Kenneth K. Tsang

**Affiliations:** HRL Laboratories LLC, 3011 Malibu Canyon Road, Malibu, CA 90265 USA

## Abstract

In this letter we report the first diamond fin field-effect transistor (diamond FinFET) without a hydrogen-terminated channel. The device operates with hole accumulation by metal-oxide-semiconductor (MOS) structures built on fins to maintain effective control of the channel conduction. Devices with 100-nm—wide fins were designed and fabricated to ensure that the channel pinched off at zero gate bias. The transfer characteristic of FinFET showed a greater than 3000 on/off ratio, successfully demonstrating the transistor behavior. Devices were characterized at room temperature and at 150 °C, showing 30 mA/mm current density at 150 °C, 35 times more than current density at room temperature. The diamond FinFET, which leverages the fin concept from the silicon industry and the material advance of diamond, enables a new class of diamond transistors for applications from digital to power and radio frequency (RF) electronics.

## Introduction

Silicon digital circuits have continuously advanced the computing front by following Moore’s Law intensely for decades, i.e., reducing energy consumption and increasing computing power by relentlessly scaling the transistor. For RF and power electronics, a different approach is needed to increase power performance since higher breakdown field is essential to increasing power density. Because of their higher breakdown field, wide-bandgap electronics have made significant impacts to the society. But after decades of development, the power density of these advanced wide-bandgap devices is approaching the fundamental limit. On the other hand, the high-power RF amplifier realm is dominated completely by vacuum-based electronics. There is a clear boundary between existing solid-state technologies and vacuum electronics^1^. This boundary is determined essentially by the breakdown field of solid-state materials. Although vacuum electronics are very powerful, they suffer from high operating voltage, complex system design, and less manufacturing scalability. An alternative solid-state approach is much more desirable if comparable power can be achieved.

Diamond has been studied for decades as a wide-bandgap semiconductor. Its superior material properties make it a perfect candidate to extend power scaling. The breakdown field in diamond is estimated to be above 10 MV/cm and was experimentally demonstrated to be around 6 MV/cm, which is much higher than GaN and SiC^2^. This fundamentally increases the power capability of diamond-based devices. More importantly, thermal conductivity in diamond reaches about 24 W/cm/K, an order of magnitude higher than other materials. This excellent thermal conduction allows diamond devices to handle much higher heat dissipation, operate at much higher junction temperature and potentially break the existing power barrier between vacuum and solid-state amplifiers. Recent decades have seen great advances in diamond research; however, the full promise of diamond-based devices has yet to be realized due to several daunting challenges. The lack of shallow level dopants is the most critical one. Several diamond transistor devices have been studied to overcome these challenges. Hydrogen-terminated FETs based on surface transfer doping^3^ showed the most significant progress. The simplicity of hydrogen termination to create a conductive channel in diamond makes it very popular in the diamond research community. However, the long-term stability of two-dimensional hole gas under high-power operation and the low mobility^4^ at high carrier concentration potentially limit device performance in the long run, although some progress was shown with surface passivation^5,6^. Taking advantage of metal-to-insulator transition (MIT), another approach utilizes a heavily boron-doped diamond thin layer as the conduction channel, creating a δ-FET^7^. In the δ layer, boron doping needs to be higher than the MIT threshold (~3 × 10^20^ cm^−3^) to supply a sufficient carrier at room temperature. Simultaneously, the sheet carrier density needs to be small enough to allow the channel to pinch off by the gate without breaking down the device. These requirements essentially limit the δ layer to be on the order of 1 nm^8^, ideally atomic layer thin. Obviously, this poses a significant challenge to material growth and surface engineering. The conductivity mobility enhancement expected in a nanometer-thin potential well was still not observed experimentally due to low carrier excitation^9^.

In this letter, we demonstrate a new diamond device concept that leverages existing diamond technologies and the latest device concept in the silicon CMOS industry. Instead of relying on an inverted channel like CMOS, we create an accumulated hole layer in diamond with MOS structure because it is very challenging to achieve channel inversion in diamond, despite some progress^10,11^ due to much wider bandgaps. MOS-induced accumulation overcomes the incomplete ionization in diamond, allowing the device to be built with a single type of dopant such as boron. However without p/n junction from the source/drain to the channel, an accumulation-based transistor inevitably suffers from large device off-leakage unless a very thin device layer is used for complete channel pinch-off by the gate^12^. With existing diamond growth technology, it is unlikely a high quality thin film, on the order of 100 nm, can be grown on a substrate without suffering from various defects^13,14^. Fortunately, other device concepts such as FinFET^15^, junctionless FET^16^, or unipolar nanowire FET^17^, developed in the silicon industry to create a fin or nanowire-type structure with the gate wrapping around the channel, provide much better channel control, especially for short-channel devices required for RF operation. Fin-like geometry was recently reported in H-terminated diamond FET^18^, but the demonstrated device requires H-termination and more importantly does not actually use fins as active device channels(the fin width is not critical to device operation). Instead, the fin-based geometry was used purely to increase conductive surface area and thus increase device current. In this letter active fin channels made in diamond were fully utilized without H-termination, enabling us to leverage thicker diamond films with much better quality and maintain the channel control at the same time for unipolar transport at the sub-micron scale. Fin geometry offers an additional degree of freedom to increase the current density by reducing the fin channel pitch and increasing the fin height, enabling a high-power-density device for RF and power electronics^18^.

## Results

**Figure 1:**
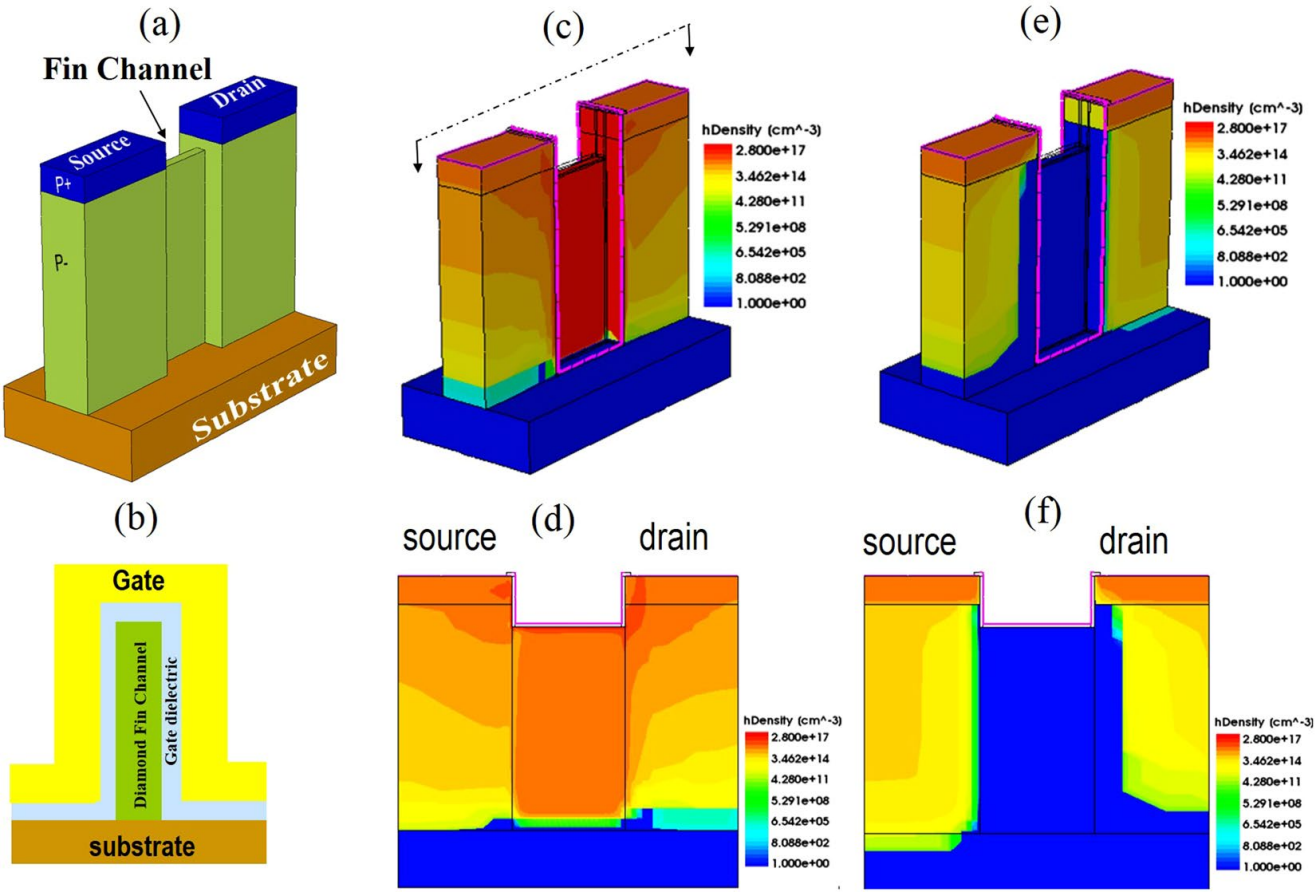
(**a**) A schematic drawing of a diamond FinFET showing source, drain, and fin channel. Heavily doped p type diamond (P+) is used in the source/drain, while lower doping is used in the channel. (**b**) The channel cross-section of the device showing the gate dielectric and gate wrapping around the fin. (**c**,**d**) Hole concentration of the device in 3D and 2D cross-sections (along the channel direction as indicated in (**c**)) when device is turned on by negative gate bias. (**e**,**f**) Hole concentration of the device in 3D and 2D cross-section when device is off at zero gate bias, indicating a completely pinched off channel.

The diamond FinFET consists of two heavily boron-doped diamond layers (P+) as source and drain sitting atop a lightly boron-doped layer (P−), Fig. 1(a). A narrow fin structure is created in between the source and drain regions as the conduction channel. The gate and gate dielectric are not drawn in Fig. 1(a). Fig. 1(b) is the cross-section of the device channel, showing the gate metal and gate dielectric wrapping around the fin channel. The whole device is built upon a semi-insulating diamond substrate to reduce substrate leakage. With negative gate bias, the diamond valence band is lifted relative to the grounded source so that the hole forms an accumulation layer originating from the source, extending through the fin channel, and to the drain. Consequently, the device is turned on. The device current density is essentially determined by the hole concentration and the hole saturation velocity for short-channel devices. Positive gate bias, on the other hand, will induce depletion regions in the channel. Depletion region width is determined by the gate metal, the gate dielectric, and the boron doping concentration of the channel through the following relation^19^:

$${V}_{g}={V}_{fb}+\frac{q{N}_{a}{W}_{dep}^{2}}{2{\varepsilon }_{s}}+\frac{q{N}_{a}{W}_{dep}}{{C}_{ox}},$$

where V_g_ is the gate bias, V_fb_ is the flat-band voltage determined by the gate metal and diamond work function, q is the single electron charge, N_a_ is the acceptor concentration, ɛ_s_ is the dielectric constant of diamond, C_ox_ is the oxide capacitance, and W_dep_ is the depletion width. The 2^nd^ term on the right represents the potential drop across the depletion region and the 3^rd^ term is the potential drop across the gate dielectric. Typically, the higher the doping concentration, the larger gate bias that is required to generate the same depletion width. To pinch off the channel at zero gate bias, the depletion width needs to be more than half the fin channel width. With an aluminum gate and 5 × 10^16^ cm^−3^ boron doping in diamond, the flat-band voltage is calculated to be −2.4 V using 1.3 eV and 4.08 eV electron affinity for diamond^20^ and Al, respectively. Using 45 nm SiO_2_ as the gate dielectric, the depletion width is calculated to be about 55 nm at zero gate bias. Hence the device is designed to have 100 nm-wide fins to ensure pinch-off at zero gate bias. To further verify this, 3D TCAD simulation in Sentaurus was carried out for a representative device with 10^19^ cm^−3^ and 5 × 10^16^ cm^−3^ boron doping in p+ and p− layers, respectively. The channel width was set to be 100 nm with Al and 45 nm SiO_2_ as gate metal and gate dielectric, respectively. The channel is 1μm long. Fig. 1(c) shows the hole concentration in 3D with the source, drain, and gate biased at 0 V, −16 V, and −10 V, respectively. Fig. 1(d) is the 2D cross-section of the device along the fin channel as indicated in Fig. 1(c). Under negative gate bias, the channel is populated with holes as shown, hence the device is turned on. The amount of charge accumulated is determined by the gate bias and the gate-to-channel capacitance. Similarly Fig. 1(e,f) show the hole distribution under zero gate bias and −16 V drain bias. The channel is completely depleted as expected from the previous calculation using the simple 1D electrostatic model. It is noted that there is a depletion region extending into the drain side. That is because the negative drain bias relative to zero gate bias pulls holes away from the drain/channel region even though the drain is doped to 10^19^ cm^−3^, which is still non-degenerated.

**Figure 2:**
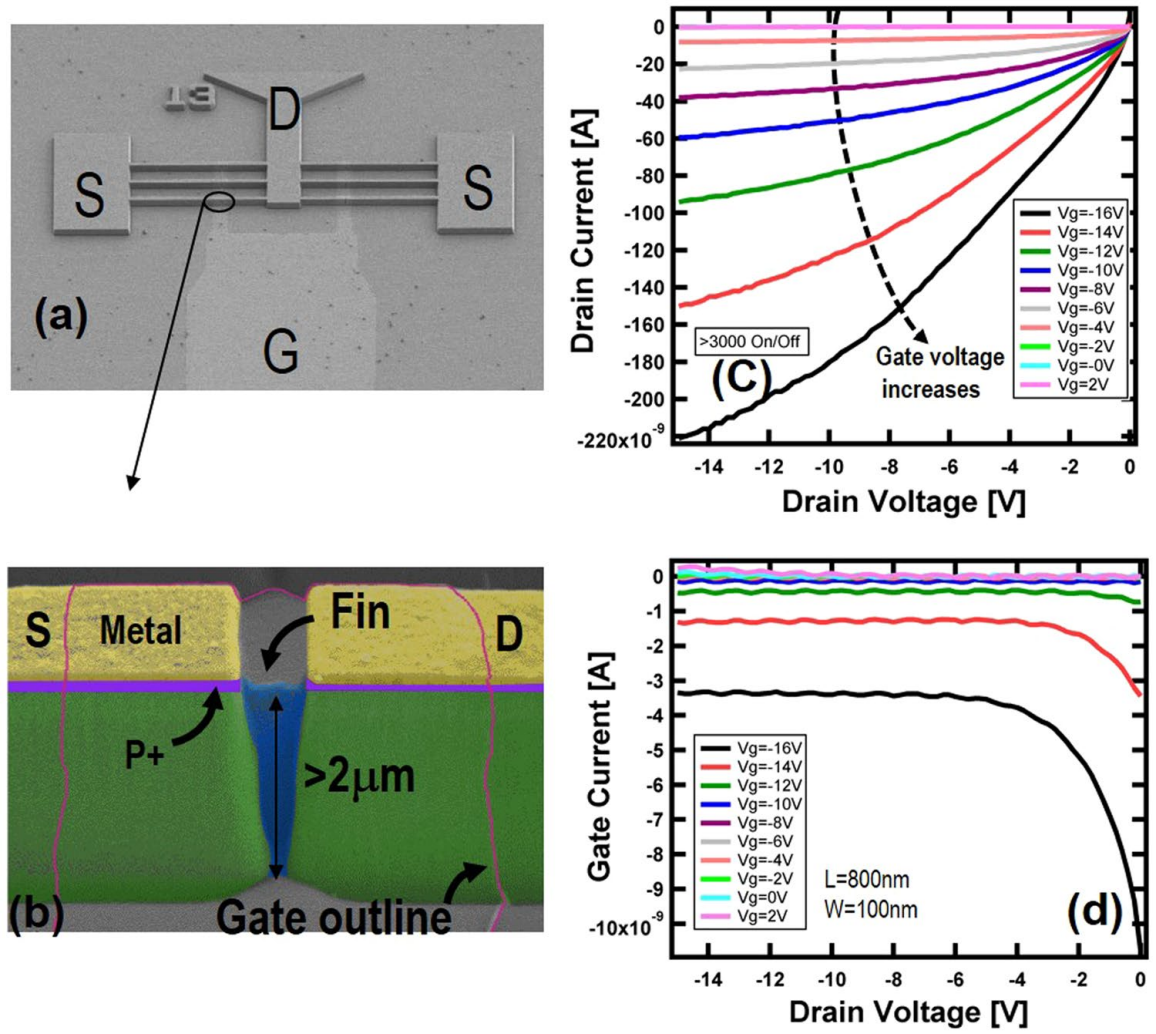
(**a**,**b**) SEM images of a diamond FinFET with source (S), drain (D), and gate (G) labeled. The device layer structure is false-colored as shown in (**b**). The gate metal boundary is drawn in pink since 100 nm Al gate metal becomes transparent under SEM. A fin channel with ~20:1 aspect ratio is formed between source and drain section, which include metal and p+/p− layers. The device channel is 800 nm long and 100 nm wide. (**c**) Device drain current versus drain voltage at different gate bias, showing >3000:1 on/off ratio (see supplementary information Fig. S1 for drain current versus gate bias). Device is pinched off at zero and 2 V gate bias. (**d**) Device gate leakage versus drain voltage at different gate biases, showing negligible leakage.

SEM images of a fabricated diamond FinFET are shown in Fig. 2(a,b). The device is a 2 × 3 finger FinFET with source (S), drain (D), and gate (G) labeled. Ti/Pt/Au was used as ohmic contact to the p+ layer as indicated in Fig. 2(b). The width of the fin channel is designed to be 100 nm. The height of the channel is determined by the thickness of the P− layer which is 2 µm, leading to ~20:1 aspect ratio on the fin. The fin is connected to the P− region underneath the p+ ohmic layer. The length of the P− channel is nominally 800 nm by design for this device, shown in Fig. 2. However, due to the sidewall slope from dry etching, the channel length is reduced significantly at the bottom of the fin after etching through the 2-µm P− film. 45 nm ALD SiO_2_ was used as gate dielectric and Al was used as gate metal. The dc transfer characteristic of the device was measured with left source, drain, and gate connected, while the right source terminal was floated. The drain current versus drain voltage under different gate biases is shown in Fig. 2(c). As expected at zero or positive gate bias (2 V), the channel is completely pinched off, hence no channel current is observed. With the increase of negative gate bias, the channel becomes increasingly conductive, as shown in Fig. 2(c). At a small drain bias, the channel is within a linear conduction regime, while it becomes saturated at a higher drain voltage bias. The maximum drain current observed was 0.22 µA, leading to larger than 3000:1 on/off ratio. The gate leakage is shown in Fig. 2(d). Only about a 10-nA gate leakage was observed at large gate/drain potential difference. The maximum current of each fin was about 70 nA, translated into 0.7 mA/mm current density assuming a 100-nm fin width(all current density in the paper are calculated with the gate width. please see supplementary information for more discussion regarding the current density calculation). These transfer characteristics clearly demonstrate the concept of diamond FinFET. The breakdown voltage of the current device is larger than the applied maximum gate voltage, −15 V. Since the gate and drain overlap, the maximum breakdown voltage is essentially determined by the gate dielectric thickness and dielectric breakdown field. 45 nm SiO_2_ would support about a 45 V breakdown voltage if we assume a 10-MV/cm breakdown field.

## Discussions

**Figure 3:**
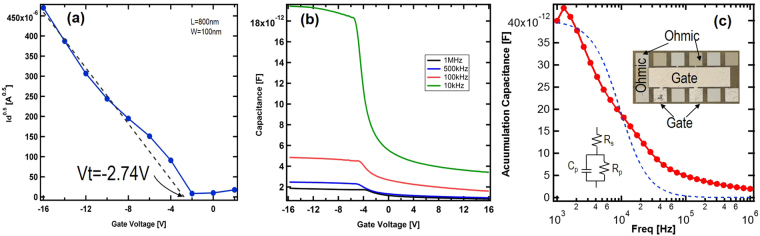
(**a**) $\sqrt{{I}_{d}}$ versus gate voltage for threshold extraction. The drain current is taken at the saturation region. The exploration indicates threshold voltage at −2.74 V. (**b**) Capacitance versus gate voltage at different frequencies for a MOS capacitor fabricated on the same chip. (**c**) The frequency dependence of maximum accumulation capacitance. The dot line is the calculated capacitance based upon a model with a serial resistor. Inset: the photo image of the MOS capacitor and the 3 component circuit model.

**Figure 4:**
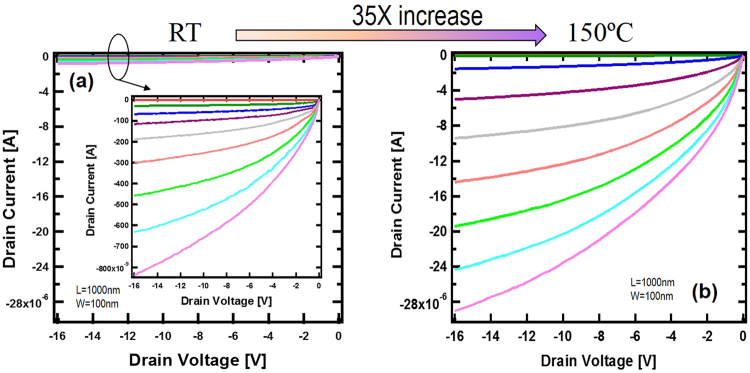
(**a**) The transfer characteristic of a diamond FinFET (9 fingers) at room temperature for gate biased from 2 to −16 V in 2 V step. The inset is the data plotted at different scale. (**b**) The transfer characteristic of the same device measured at 150 °C.

The device threshold voltage can be determined by the linear extrapolation method in the saturation region to avoid the impact of series resistance^21^. As shown in Fig. 3(a), the threshold voltage was measured at about −2.74 V, close to the flat-band voltage calculated by the band alignment. This is consistent with capacitance vs. gate voltage (CV) measurements on MOS capacitors fabricated on the same chip, as shown in Fig. 3(b). The photo image of the MOS capacitor is shown in Fig. 3(c) inset. The diamond substrate was grounded, hence at large negative gate biases, holes formed an accumulation layer, showing maximum capacitances. However, the measurement of maximum capacitance at accumulation regime for different frequencies from 1 kHz to 1 MHz, shown in Fig. 3(c), clearly indicates the impact of series resistance: with increased measuring frequency, maximum accumulation capacitance drops. Even at 10 kHz in Fig. 3(b), maximum capacitance only reaches 19 pF for a 50050-μm^2^ MOS capacitor, about half of the theoretical value (38 pF) based upon the SiO2 thickness. Only at very low frequencies can the theoretical value be reached due to much less impact of the series resistance. This is consistent with other frequency-dependent studies for diamond MOS capacitors^10^. Taking the series resistance into account, the maximum accumulation capacitance at various frequencies can be calculated as ${C}_{pm}=\frac{{C}_{p}}{1+{({R}_{s}\omega {C}_{p})}^{2}}$ where C_pm_, Cp, Rs and ω are measured maximum capacitance, intrinsic maximum capacitance, series resistance and measurement frequency, respectively. The equivalent circuit is shown in Fig. 3(c). At accumulation region, the parallel conductance (1/R_p_) is much smaller than the conduction of the capacitor. Hence the circuit is reduced to two components at accumulation regime. As shown in Fig. 3(c), the model and measured data show good agreement on the general trend of frequency dependency. The disagreement might originate from the interface charge on non-ideal diamond/dielectric interface. The ohmic contact in our devices is formed on nominally 10^19^ cm^−3^ boron-doped diamond. Although the doping in contact regions is much higher than that in the channel, it is still a much-less-than-typical 2–5 × 10^20^ cm^−3^ doping level needed for metal-to-insulator transition in diamond. Due to incomplete ionization, only less than 0.1% boron is activated at room temperature, leading to much larger contact resistance. This explains the frequency dependency in MOS CV measurement. To further understand this and explore the device’s capability, a similar diamond FinFET was measured at higher temperature. Fig. 4(a,b) show the measured IV at room temperature (RT) and 150 °C respectively. They were drawn in the same scale. Fig. 4(a) inset is the RT data redrawn in a different scale to show the transfer characteristics. At room temperature the maximum drain current is about 838 nA for −16 V bias on gate and drain. By operating the device at elevated temperatures, the current is increased by a factor of 35 to about 29 μA with the same bias. Through a TLM structure, the ohmic contact resistances at RT and 150 °C were extracted to be 493 ohm*mm and 47 ohm*mm, respectively. For 10^19^ cm^−3^ boron doping, the activation efficiency was calculated to be 5 × 10^−4^ at room temperature. At 150 °C the activation efficiency increased to about 5 × 10^−3^. This is consistent with the conduction current increase as shown in Fig. 4 and the reduction of the contact resistance.

In conclusion, we successfully demonstrated a diamond FinFET with more than 3000 on/off ratio. The threshold voltage and MOS capacitance measurements clearly show hole accumulation in the device. The 30 mA/mm maximum current density was observed at 150 °C. The relatively low current density was mainly limited by high ohmic contact resistance due to incomplete ionization. The observation of higher conductivity at elevated temperatures indicates the potential of diamond FinFETs for high-temperature environments. It is reasonable to expect that the contact resistance can be improved significantly by incorporating more boron doping because higher boron doping beyond metal-to-insulator transition has been demonstrated in diamond for superconducting applications. For practical applications in power or RF electronics, diamond transistors need to provide at least 1 A/mm current density to be competitive. The cutoff frequency of diamond FinFET also needs to reach 100 GHz to be useful for RF applications at Ka band. Certainly, there are challenges to continuously improving the device performance through ohmic, gate dielectric engineering, however, this new diamond transistor device design, fully leveraging the existing technologies, represents a paradigm shift for future diamond research ranging from digital to RF electronics.

## Methods

The starting sample was a (100) 3 × 3 mm undoped diamond substrate with an epitaxially grown p+/P− bilayer on top. The p+ layer was patterned and dry etched to define the ohmic area and also to expose the channel area. Ti/Pt/Au was evaporated to form a good ohmic contact after 525 °C annealing in argon gas. E-beam lithography and O_2_ plasma dry etching were used subsequently to form 100-nm wide and 2-μm tall fins. SiO_2_ gate dielectric was deposited by atomic layer deposition at 200 °C. To conformably wrap the gate around the sidewalls of the fins, Al metal was sputtered with photoresist in place, then the metal was lifted off. Only 100 nm Al was used to ensure successful liftoff by sputtering. Finally the ohmic contact pads were open with wet etching.

## Electronic supplementary material

Supplementary Information
