# Supplementary material for: Diamond FinFET without Hydrogen Termination
Source: Sci Rep. 2018 Feb 15;8:3063. doi: 10.1038/s41598-018-20803-5 (PMC5814511; doi:10.1038/s41598-018-20803-5)
Supplement: Supplementary file 1 — Supplementary Information [file 41598_2018_20803_MOESM1_ESM.docx]

**Supplementary Information**

**Diamond FinFET without Hydrogen Termination**

Biqin Huang*, Xiwei Bai, Stephen K Lam, Kenneth K Tsang

HRL Laboratories LLC, 3011 Malibu Canyon Road, Malibu, CA, 90265

* bhuang@hrl.com

1. Drain current versus gate voltage

Supplementary Figure S1 the drain current versus the gate voltage at high (-12V) and low (-3V) drain bias for a device with 800nm long and 100nm wide channel. The relative high off current for high drain bias is caused by gate leakage current due large drain/gate potential difference. The off current for low drain bias is basically the noise floor.

2. Current density calculation for FinFET

Because of fin geometry, the calculation of current density is more complicated than a typical planar device. Assuming the height H and the width W for the gate, the channel current can be either normalized by the physical width W or by the effective channel width 2H+W. To study the transport physics of the device, it is necessary to use 2H+W for current density calculation, since it represents the true width of the current transport path. Using this normalization will give correct estimation for other useful information such as mobility. However, in this paper, the current density is calculated by using the gate width W. This is to facilitate the comparison between diamond FinFET and other wide band gap lateral RF transistors. For RF/power applications the device footprint is important. Hence, the relevant current density and other specs are typically calculated against the footprint (normally against the device width which is typically the channel width). In FinFET, the fin height introduces an additional degree of freedom to increase the effective channel width, but it doesn’t change the device surface area which is more relevant in device applications. This is actually an advantage of diamond FinFET relative to planar devices, enabling the increase of current without increasing the surface area. By doing this, it essentially increases the current density (against device width). Please note in practical devices, the pitch of the fin channel is also critical. It will affect the current density calculation for comparison. Since this paper is about the concept demonstration, we did not include this factor.
